# JUL1, Ring-Type E3 Ubiquitin Ligase, Is Involved in Transcriptional Reprogramming for ERF15-Mediated Gene Regulation

**DOI:** 10.3390/ijms24020987

**Published:** 2023-01-04

**Authors:** Junna Kawaguchi, Kaito Hayashi, Yoshitake Desaki, Abdelaziz Ramadan, Akira Nozawa, Keiichirou Nemoto, Tatsuya Sawasaki, Gen-ichiro Arimura

**Affiliations:** 1 Department of Biological Science and Technology, Faculty of Advanced Engineering, Tokyo University of Science, Tokyo 125-8585, Japan; 2 Proteo-Science Center, Ehime University, Matsuyama 790-8577, Japan; 3 Iwate Biotechnology Research Center, Kitakami 024-0003, Japan

**Keywords:** abscisic acid, *Arabidopsis thaliana*, ERF15, jasmonate, JAV1, JAV1-associated ubiquitin ligase1 (JUL1), *Spodoptera litura*

## Abstract

JAV1-associated ubiquitin ligase 1 (JUL1) is a RING-type E3 ubiquitin ligase that catalyzes ubiquitination of JAV1, a jasmonate signaling repressor, in *Arabidopsis thaliana* in response to herbivore attack. Here we present a new insight into the nature of JUL1 as a multi-targeting enzyme for not only JAV1 but also transcription factors (TFs) screened using in vitro and in vivo protein interaction assays. Reporter assays using protoplasts showed that the JUL1-interacting TFs (JiTFs), including ERF15, bZIP53 and ORA59, were involved in transcriptional activation of jasmonate-responsive *PDF1.2* and abscisic acid-responsive *GEA6*. Likewise, assays using mutant plants suggested that the 3 JiTFs were indeed responsible for transcriptional regulation of *PDF1.2* and/or *GEA6*, and ERF15 and ORA59 were substantially responsible for the anti-herbivore trait. In vitro protein ubiqutination assays showed that JUL1 catalyzed ubiqutination of JAV1 but not any of the TFs. This was in accord with the finding that JUL1 abolished JAV1′s interference with ERF15 function, according to the reporter assay. Moreover, of great interest is our finding that ERF15 but not bZIP53 or ORA59 serves as a scaffold for the JAV1/JUL1 system, indicating that there is narrow selectivity of the transcriptional reprogramming by the JAV1/JUL1 system.

## 1. Introduction

Transcriptional reprogramming plays important roles in the jasmonate (JA)-mediated defense response against herbivores in higher plants. Plants rely on this system for the tradeoff between plant growth and defense modes when they are either in the steady state or suffer from biotic stresses [1]. The jasmonate ZIM-domain (JAZ) family members are major transcriptional repressors of JA-responsive genes that act by binding and inhibiting MYC transcription factors (TFs) in the steady state of *Arabidopsis thaliana* (Arabidopsis). In response to herbivore damage, plants upregulate the biosynthesis of Ile-conjugated JA (JA-Ile), which binds to SCF (COI1) ubiquitin ligase to cause degradation of JAZ proteins via the ubiquitin-26S proteasome system (UPS), thereby leading to the release of MYCs to activate the transcription of JA-responsive genes [2,3]. In brief, Arabidopsis exhibits a steady/defense mode-switching system that coordinates the transcriptional status of JA-responsive genes according to the absence or presence of JAZ repressors.

Like JAZ repressors, jasmonate-associated VQ-motif gene1 (JAV1) forms a complex with JAZ8 to repress WRKY TFs (e.g., WRKY51), in the steady state of Arabidopsis [4,5]. However, in response to herbivore damage, JAV1 is degraded through UPS catalyzed by JAV1-associated ubiquitin ligase1 (JUL1), a RING-type E3 ubiquitin ligase, thereby causing the release of WRKYs to activate transcription of the *plant defensin1.2* (*PDF1.2*) gene [6]. Intriguingly, JUL1 also functions in abscisic aid (ABA)-promoted stomatal closure. During plant draught stress, JUL1 interacts with microtubules and causes microtube depolymerization in guard cells, resulting in stomatal closure and increased drought tolerance of the plant [7]. Thus, JUL1 is localized ubiquitously in cytosolic microtubules and the nucleus and plays multi-functional roles in resistance against both biotic and abiotic stresses, probably by switching substrate targets [7].

Notably, the JAV1/JUL1 system has been considered to contribute specifically to transcriptional programming of WRKY-mediated gene activation in the nucleus, as VQ family proteins, including JAV1 (VQ22), have been assumed to interact specifically with the WRKY domain of groups I and IIc WRKYs [8]. However, this assumption was disproven by findings in AlphaScreen-based assays carried out to analyze the potential interactions of TFs with JAV1 [6] as well as JUL1 (this study). These assays showed that JAV1 interacted predominantly with a suite of WRKY family proteins (e.g., WRKY28 and WRKY48) [6], but, intriguingly, JUL1 was able to interact with other TFs, including MYB, apetala2/ethylene response factor (AP2/ERF), and basic leucine-zipper (bZIP) (see the Results section; Table S1). Considering these facts collectively, we hypothesized that (1) JUL1 interacts with not only JAV1 but also an array of TFs and (2) the JAV1/JUL1 system works to regulate not only WRKYs but also other families of TFs. Thus, in the current study, to test these hypotheses, we explored whether JUL1-interacting transcription factors (denoted as “JiTFs”) are functionally involved in transcriptional reprogramming of defense-related genes to fine tune the anti-herbivore trait against *Spodoptera litura*, a generalist herbivore that feeds on foliage and fruit of several plant taxa, including agricultural crops [9]. For this, we focused on the nature of transcriptional regulation of the JA-responsive plant defensin gene *PDF1.2* [10] and ABA-responsive *GEA6* (UDP-D-glucuronate 4-epimerase 6) [11]. JAs act extensively as master regulators for anti-herbivore activity [12], and ABA is known to act as a coordinator of JA signaling, at least in Arabidopsis, during herbivory [13]. Finally, we present a model in which JAV1/JUL1 coordinate transcriptional machinery that specifically works in ERF15-mediated plant defense responses. 

## 2. Results

### 2.1. Transcriptional Activation of PDF1.2 and GEA6 Promoters through JiTFs

**Figure 1:**
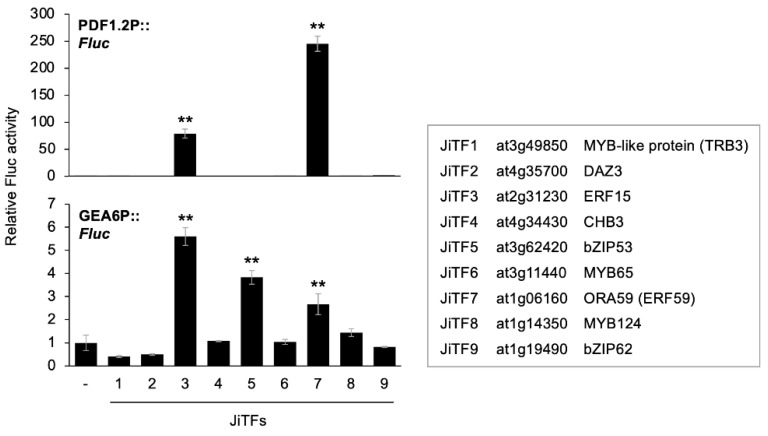
Transcriptional activity of JiTFs. Transient activation of a firefly luciferase (Fluc) reporter gene under the control of *PDF1.2* promoter (PDF1.2P) or *GEA6* promoter (GEA6P) following expression with or without (−) *JiTFs* in *Arabidopsis thaliana* protoplasts. Data represent the mean and standard error (*n* = 5). Data marked with asterisks are significantly different from those obtained without *JiTFs*, based on an ANOVA with Holm’s sequential Bonferroni post hoc test (**, *p* < 0.01).

As described above, we assessed in vitro interaction between biotinylated recombinant JUL1 protein and 566 FLAG-conjugated TF proteins, using the AlphaScreen system. The resultant luminescence intensities showed that an array of TF proteins (denoted as JUL1-interacting transcription factors [JiTFs]) from various TF families were able to interact with JUL1, among which 26 TFs showed especially strong interaction ability (more than 100-fold stronger when compared with *Escherichia coli* dihydrofolate reductase) (Table S1). Intriguingly, WRKY family members were not listed among these 26 JiTFs, except for WRKY33, whereas members of other TF families, including MYB, AP2/ERF, and bZIP, were included (Table S1), in contrast to the reported interactions of JAV1 with TFs [6]. We then focused on the 10 JiTFs found to interact most strongly with JUL1, except for WRKY33, whose cDNA failed to be amplified by subsequent polymerase chain reaction (PCR)-based cloning (see Figure 1). 

To assess the in vivo function of the resultant 9 JiTFs, each of them was coexpressed with a firefly luciferase (Fluc) reporter gene under the control of the 5′ flanking region of the *PDF1.2* gene (PDF1.2P) or *GEA6* gene (GEA6P) in protoplasts prepared from Arabidopsis leaves. Protoplasts coexpressing PDF1.2P::*Fluc* with *JiTF3* (*ERF15*) or *JiTF7* (*octadecanoid-responsive arabidopsis59* [*ORA59*], a member of the ERFs) showed dramatically increased levels of Fluc activity, compared to the level in protoplasts expressing PDF1.2P::*Fluc* alone (Figure 1). When protoplasts concomitantly expressed GEA6P::*Fluc* and each of *JiTF3* (*ERF15*), *JiTF5* (*bZIP53*) or *JiTF7* (*ORA59*), moderately increased levels of Fluc activity were observed, compared to that in protoplasts expressing PDF1.2P::*Fluc* alone (Figure 1). Taking these findings all together, we concluded that the 3 TFs (ERF15, bZIP53 and ORA59) were trans-activators of PDF1.2P and/or GEA6P, and we further examined them by the following analyses.

### 2.2. Anti-Herbivore and JA/ABA Responses Mediated by ERF15, bZIP53 and ORA59 

**Figure 2:**
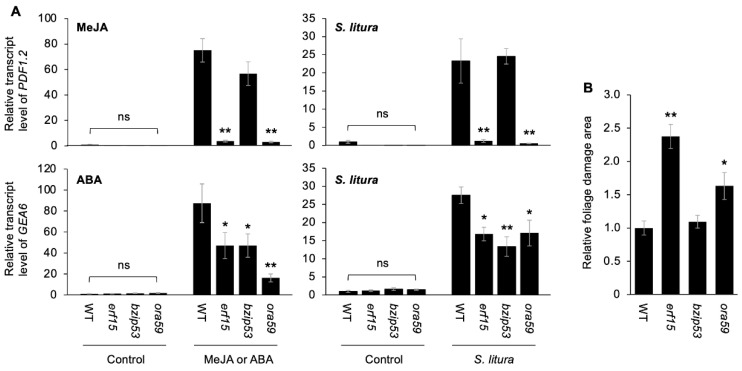
Anti-herbivore traits of *JiTF*-deficient mutant plants. (**A**) Relative transcript levels of *PDF1.2* and *GEA6* in leaves of wild-type (WT) *Arabidopsis thaliana* plants and T-DNA insertion mutant plants, corresponding to the respective JiTFs (JiTF3 [*erf15*], JiTF5 [*bzip53*], and JiTF7 [*ora59*]), were exposed to methyl jasmonate (MeJA) for 12 h, abscisic acid (ABA) for 6 h, or *Spodoptera litura* larvae for 24 h. Plants with application of aqueous solution (0.1% ethanol) alone for 12 h and for 6 h and uninfested healthy plants served as control for MeJA, ABA and *S. litura*-exposed plants, respectively. (**B**) Foliage damage areas of mutant plants, relative to those of WT plants, were assessed after incubation with *S. litura* larvae for 24 h. Data represent the mean and standard error (*n* = 6 and 20 for (**A**) and (**B**), respectively). Data marked with an asterisk(s) are significantly different from those in WT among each data set, based on an ANOVA with Holm’s sequential Bonferroni post hoc test (**, *p* < 0.01; *, 0.01 ≤ *p* < 0.05). ns, not significant.

To understand in more detail the in planta function of ERF15, bZIP53 and ORA59, T-DNA insertion mutant lines (*erf15*, *bzip53* and *ora59*) were studied. These mutants exhibited lower transcript levels of the corresponding genes *ERF15*, *bZIP53* and *ORA59* (Figure S1). Transcript levels of the JA-responsive gene *PDF1.2* elicited in leaves of *erf15* and *ora59* plants by methyl jasmonate (MeJA) treatment and damage by *Spodoptera litura* larvae were much lower than those in WT leaves (Figure 2A). Likewise, transcript levels of the ABA-responsive gene *GEA6* elicited in leaves of *erf15*, *bzip53* and *ora59* plants by methyl jasmonate (MeJA) treatment and the herbivory were lower than those in WT leaves (Figure 2A). These defects of transcription responses in the mutants resulted in increased foliage damage in *erf15* and *ora59* but not *bzip53* plants, when compared to that in WT (Figure 2B).

### 2.3. Transcriptional Reprogramming with JAV1, JUL1 and JiTFs

**Figure 3:**
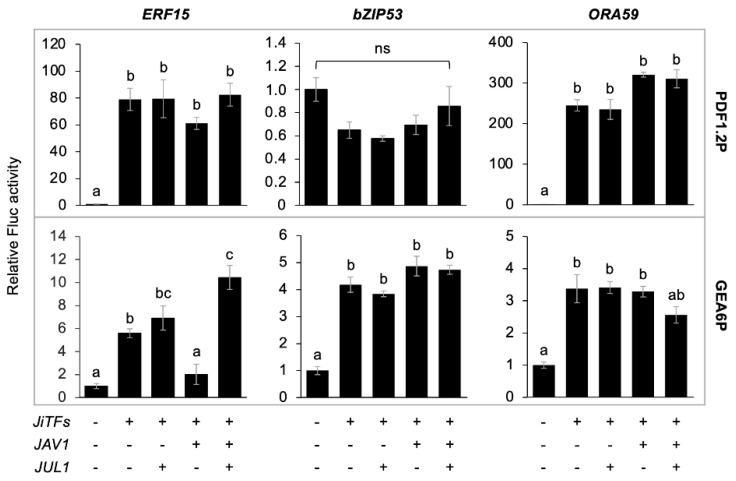
Modulation of JiTF1-mediated *PDF1.2* promoter (PDF1.2P) and *GEA6* promoter (GEA6P) activities via JAV1/JUL1. Transient activation of a firefly luciferase (*Fluc*) reporter gene under the control of PDF1.2P and GEA6P following expression with (+) or without (−) *JiTFs* (*JiTF3* [*ERF15*], *JiTF5* [*bZIP5*] and *JiTF7* [ORA59]), *JAV1* or *JUL1* in *Arabidopsis thaliana* protoplasts. Data represent the mean and standard error (*n* = 5). The means indicated by different small letters are significantly different among data of each day, based on an ANOVA with post hoc Tukey’s HSD (*p* < 0.05). ns, not significant.

Since JUL1 was able to interact directly with JiTFs (Table S1), we assumed that JUL1 might serve as repressor of not only JAV1 but also JiTFs in the transcriptional control of *PDF1.2* and *GEA6*. To test this hypothesis, PDF1.2P::*Fluc* or GEA6P::*Fluc* was expressed concomitantly with *JUL1* and each of the *JiTFs* (*ERF15*, *bZIP53* and *ORA59*) in protoplasts. The resultant Fluc activity levels were not affected when *JUL1* was coexpressed with each of the *JiTFs*, in comparison to when each of the *JiTFs* alone was expressed (Figure 3). In contrast, when PDF1.2P::*Fluc* or GEA6P::*Fluc* was expressed concomitantly with *JAV1* and each of the *JiTFs* in protoplasts, the ERF15-promoted GEA6P::*Fluc* activity levels declined, compared to those without *JAV1*. Then, concomitant expression of *JUL1* caused recovery of Fluc activity levels. Likewise, the PDF1.2P-promoted Fluc activity levels with *ERF15* expression were marginally decreased when *JAV1* was coexpressed and restored by concomitant expression of *JUL1* (*p* > 0.05). Moreover, none of the Fluc activities with *bZIP53* or *ORA59* expression was modulated when *JAV1* or *JAV1* + *JUL1* was coexpressed. Thus, contrary to our hypothesis, these results suggested that JUL1 serves as a repressor of JAV1 only when JAV1 exhibited the sufficient deterrence to the selective JiTFs such as ERF15.

### 2.4. Not Enhanced Ubiquitination of JAV1 by JUL1

**Figure 4:**
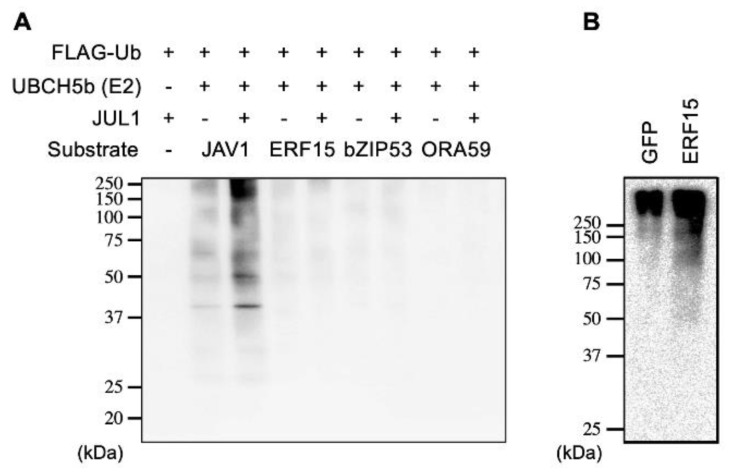
In vitro JUL1 ubiquitination activity on JAV1 and JiTFs (JiTF3 [ERF15], JiTF5 [bZIP5] and JiTF7 [ORA59]) as substrates. (**A**) Biotinylated JiTFs and JAV1 proteins were incubated with FLAG-tagged ubiquitin (FLAG-Ub), UBCH5b (E2) and AGIA-tagged JUL1. (**B**) Likewise, biotinylated JAV1 proteins were incubated with FLAG-Ub, UBCH5b, and AGIA-tagged JUL1 in the presence of AGIA-tagged GFP or ERF15. The incubated reaction mixtures were immunoprecipitated using streptavidin-linked magnetic beads, subjected to SDS-PAGE, and probed with an anti-FLAG-HRP (FLAG-Ub) antibody. The smeared signals indicate possible ubiquitinated substrate proteins.

The findings shown in Figure 3 raised another question, namely, whether JUL1 was able to ubiquitinate JiTFs, as it does JAV1. To test this, we next assessed the in vitro ubiquitination activity of JUL1 on biotinylated JAV1 (Bio-JAV1) or each of the biotinylated JiTF proteins (Bio-ERF15, Bio-bZIP53 and Bio-ORA59) as substrates, with FLAG-tagged ubiquitin (FLAG-Ub), UBCH5b (E2) and AGIA-tagged JUL1 proteins (AGIA-JUL1). Immunoprecipitation with anti-biotin and subsequent anti-FLAG antibody detection revealed that AGIA-JUL1 catalyzed ubiquitination of Bio-JAV1 protein but not of any biotinylated JiTF proteins (Figure 4A). Moreover, Bio-JAV1 protein was incubated with FLAG-Ub, UBCH5b, and AGIA-JUL1 proteins in the presence of either AGIA-tagged ERF15 protein (AGIA-ERF15) or green fluorescent protein (GFP) protein (AGIA-GFP) serving as control. We found that Bio-JAV1 was ubiquitinated similarly by JUL1 when it was incubated with AGIA-ERF15 and when it was incubated with AGIA-GFP (Figure 4B), indicating that ERF15 was not likely to serve as an enhancer of ubiquitination of JAV1.

### 2.5. In Planta Complex Composed of JAV1, JUL1 and ERF15

**Figure 5:**
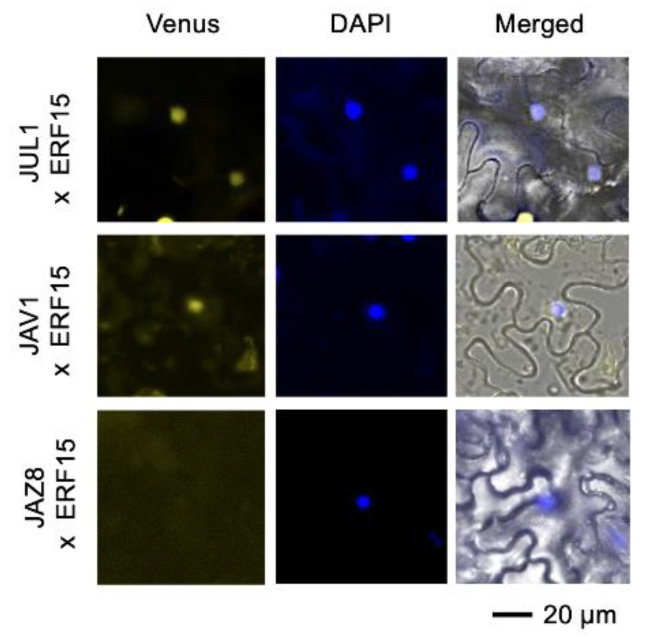
Bimolecular fluorescence complementation analysis of *in planta* interaction of JUL1, JAV1 or JAZ8 fused to the N-terminal fragment of Venus with ERF15 fused to the C-terminal fragment of Venus in *Nicotiana benthamiana* leaf cells. The photographs with reconstructed Venus signal, DAPI (4′,6-diamidino-2-phenylindole) fluorescence, and the merged image with bright field are shown.

Finally, in order to confirm the in planta interaction of ERF15 with JUL1 and JAV1, we performed bimolecular fluorescence complementation (BiFC) assays. Concomitant expression of JUL1 or JAV1 fused to the N-terminal fragment of Venus (nVenus) and ERF15 fused to the C-terminal fragment of Venus (cVenus) in *Nicotiana benthamiana* leaf cells resulted in fluorescent signals from the reconstructed YFP proteins in the nuclei (Figure 5). The same held true for expression of JUL1 or JAV1 fused to nVenus, concomitantly with bZIP53 or ORA59 fused to cVenus (Figure S2). Moreover, whereas JAV1-interacted WRKY51 interacted with JAZ8 repressor as reported [5], ERF15 was not able to interact with JAZ8 (Figure 5), indicating different constituent molecules in the JAV1/ERF15 complex compared to the JAV1/WRKY51 complex.

## 3. Discussion

As described above, the JAV1/JUL1 system had been believed to work only for transcriptional reprogramming of WRKY family TFs, as JAV1 is a member of the VQ motif-containing proteins (VQ proteins) that interact specifically with WRKYs to repress their function [14]. However, findings in the current study demonstrated that the JAV1/JUL1 system is more extensively involved in the regulation of multiple JiTFs during herbivore attack. Notably, the function of the JAV1/JUL1 system was specifically pronounced in the case of ERF15-promoted transcriptional control (Figure 3).

**Figure 6:**
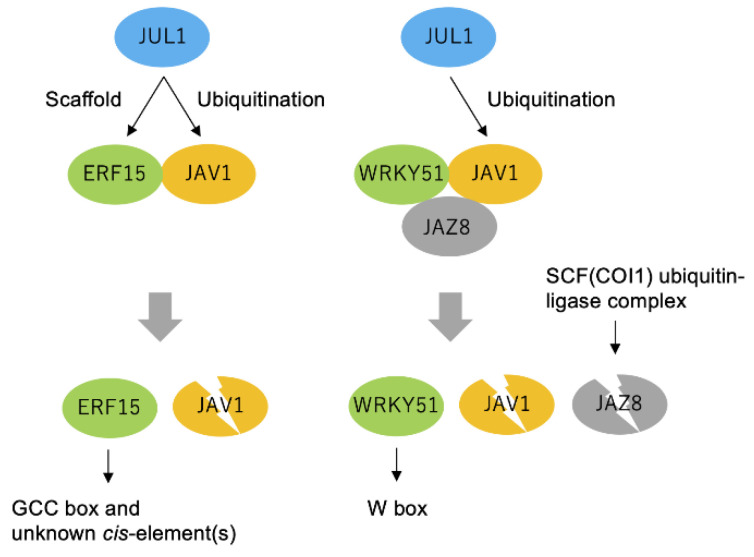
A possible model of the two ways of JUL1-catalyzed JAV1 ubiquitination when ERF15 or WRKY is captured by JAV1 in *Arabidopsis thaliana*. In brief, in the steady state of the plant, the JAV1/ERF15 complex has different constituent molecules from the JAV1/WRKY51 complex which harbors JAZ8. In response to herbivory, JUL1 starts ubiquitination of JAV1 with a scaffold on ERF15, resulting in degradation of JAV1 via the ubiquitin-26S proteasome system. On the other hand, the JAV1/WRKY51/JAZ8 complex is decomposed with degradation of JAV1 and JAZ8 via JUL1 and SCF(COI1) ubiquitin-ligase complex, respectively. The consequent releases of ERF15 and WRKY51 act at the respective specific *cis*-elements, leading to gene regulation.

The nature of JAV1 ubiquitination catalyzed by JUL1 in the JAV1/JUL1/ERF15 complex would be distinct from that catalyzed in an earlier reported complex composed of WRKY (WRKY51), JAV1 and JUL1 [5] (Figure 6). This is supported by the facts that (i) JUL1 interacts preferably with non-WRKYs (except some WRKYs such as WRKY33) (Table S1) and (ii) ERF15 does not interact with JAZ8 (Figure 5), unlike WRKY51 [5]. In other words, it appears that there are different constituent molecules between these 2 complexes (Figure 6). Presumably, unlike WRKYs exhibiting strong affinity for JAV1 [6], JUL1 may need to scaffold on ERF15 to achieve effective ubiquitination of JAV1, although in vitro ubiquitination assays did not show significantly increased ubiquitination of JAV1 in the presence of ERF15 (Figure 4). However, since the total detected, ubiquitinated JAV1 proteins in the relevant assays should include a large amount of free JAV1 as well as JAV1 interacting with proteins other than ERF15, the actual function of the scaffolding of JUL1 specifically on ERF15 remains to be clarified.

Except for the representative model case of the JAV1/JUL1/ERF15 complex, the nature of possible interactions of JUL1 with the other JiTFs (Table S1) remains to be clarified. This is because bZIP53 and ORA59 were able to activate PDF1.2P and/or GEA6P but these promotions were not modulated upon coexpression with either JAV1 or JUL1 in the respective reporter assays (Figure 3). Of course, it is possible that most JiTFs may function specifically at promoters other than PDF1.2P and GEA6P. Alternatively, the JAV1/JUL1 system may require functionalization by cofactors, for example, like the functionalization shown in the plant immune system, in which phosphorylation of RBOHD and NPR1 is required to enhance UPS-based protein degradation [15,16]. However, it is also possible that phosphorylation-based protein modifications are even required to interfere with molecular interactions [17] and UPS-based protein degradation [18].

Indeed, compared to the transcriptional regulation of *PDF1.2* by ERF13, the function of ERF13 at the *GEA6* promoter is puzzling. Although PDF1.2P (~1000 bp upstream promoter region of *PDF1.2*) has a single GCC box (general ERF-binding *cis*-element [19] [AGCCGCC]), GEA6P (~1273 bp upstream promoter region of *GEA6*) does not. Nevertheless, ERF13 is certainly involved in transcriptional activation of *GEA6* in planta (Figure 1). In fact, some of the AP2/ERF transcription factors have been shown to bind to non-GCC box (O box ([NC/GT]CGNCCA) [20], DRE box ([A/G]CCGAC) and CE1 (CAC[C/A]G) [21]). Those *cis*-elements are, however, also absent within GEA6P, implying that there are other variable types of *cis*-elements for binding of AP2/ERF family members. Moreover, it cannot be excluded that JiTFs other than the ERF15, bZIP53 and ORA59 focused on in this study were not able to activate either PDF1.2P or GEA6P. This indicates that they serve for regulating other sets of genes and the JAV1/JUL1 system may manage transcriptional reprogramming in such not yet characterized systems.

## 4. Materials and Methods

### 4.1. Primers 

Primers used for all of the polymerase chain reactions (PCR) in this study are listed in Table S2.

### 4.2. Cell-Free Protein Synthesis, AlphaScreen Assay and Immunoblotting

The full-length open reading frames (ORFs) of *JUL1* and of each cDNA clone of 566 Arabidopsis TFs whose cDNAs were successfully amplified by PCR from the full-length library [22] were inserted into the Gateway (GW) vectors pEU-6×His-bls-GW (bls; biotin ligation site) and pEU-GW-FLAG, respectively. Cell-free protein synthesis and AlphaScreen-based protein-protein interaction assays were carried out according to the methods described previously [6,23]. All data represent the average of three independent experiments, and the background was controlled using DR from *Escherichia coli*. Total proteins derived from the cell-free protein synthesis system were subjected to 10% SDS-polyacrylamide gel electrophoresis (PAGE) and immunoblotted with anti-AGIA HRP-linked antibody [23], anti-FLAG HRP-linked antibody (FUJIFILM Wako Pure Chemical Corporation, Ltd., Osaka, Japan) or anti-biotin HRP-linked antibody (Cell Signaling Technology, Beverly, MA, USA) (Figure S2). The immunoblot membranes were soaked with Immobilon Western Chemiluminescent HRP Substrate (Merck Millipore Ltd., Darmstadt, Germany) and the signals were detected with an ImageQuant LAS-4000 imaging system (GE Healthcare, Buckinghamshire, UK) (Figure S3).

### 4.3. Plants

*Arabidopsis thaliana* (Arabidopsis) ecotype Col-0, its T-DNA insertion mutant lines (*erf15* [GK-830E01], *bzip53* [SALK_004683] and *ora59* [GK_061A12]), and transgenic line overexpressing JUL1 (JUL1-OX2) [6] were grown in a growth chamber at 22 ± 1 °C with a photoperiod of 14 h (80 µE m^–2^ s^–1^). Likewise, *Nicotiana benthamiana* plants were grown in soil in climate-controlled rooms at 24 ± 1 °C with a photoperiod of 12 h (80 µE m^–2^ s^–1^) and at 24 ± 1 °C with a photoperiod of 16 h (80 µE m^–2^ s^–1^). The individual plants were grown in single plastic pots for 4 weeks. The potted, 4-week-old Arabidopsis plants and 6-week-old *N. benthamiana* plants were used for subsequent analyses.

We used the T3 generation of homozygous T-DNA lines of Arabidopsis plants after screening by PCR tests (http://signal.salk.edu/tdnaprimers.2.html (accessed in 1 December 2022)).

### 4.4. Chemical and Herbivore Treatment

For chemical treatment, potted plants were evenly sprayed with 1 mL of MeJA (FUJIFILM Wako Pure Chemical Corporation, Ltd.; 0.5 mM) and 2-*cis*,4-*trans*-abscisic acid (Sigma-Aldrich, St. Louis, MO, USA; 0.1 mM) in aqueous solution (0.1% ethanol). These treated plants and plants treated with 0.1% ethanol solution (serving as control) were incubated in climate-controlled rooms at 22 ± 1 °C under the light condition 80 µE m^–2^ s^–1^ for up to 12 h (MeJA) or 6 h (ABA). For herbivore treatment, a potted plant was incubated with a single larva of *S. litura* (1.8-2.0 mg) at 24 ± 1 °C at a light intensity of 80 µE m^–2^ s^–1^ for 24 h. The leaves exposed to the above chemicals or herbivore were harvested and stored at −80 °C for gene expression analysis (see below), or all of the *S. litura*-damaged leaves were scanned, and the total leaf area and the consumed leaf area were determined using ImageJ.

### 4.5. Protoplast Preparation and Transfection

The full-length ORFs of *JUL1*, *JAV1*, and each of the transcription factors (*TFs*) were, respectively, cloned into the p35SΩ-GW-V5-NOST vector (cauliflower mosaic virus 35S promoter [35SP]::Ω sequence [translation enhancer]:: GW::V5::*nopaline synthase* terminator [NOST]), p35SΩ-GW-3FLAG-NOST vector (35SP::Ω sequence::GW::3×FLAG:: NOST), and p35S-GW-4MYC-NOST vector (35SP::GW:: 4× Myc::NOST). The promoter region (40–1000 bp upstream region) of *PDF1.2* (PDF1.2P) and the promoter region (1–1273 bp upstream from start codon) of *GEA6* (GEA6P; [24] were cloned, and inserted in front of the *firefly luciferase* (*Fluc*) reporter gene::NOST cassette in the pMA cloning vector (Thermo Fisher Scientific, Waltham, MA, USA).

Protoplast cells were isolated from approximately 50 mature Arabidopsis WT leaves as previously described [25,26]. Finally, 2.0 × 10^5^ protoplasts mL^−1^ were prepared. Vector transformation of each sample was performed independently after isolation of the protoplasts as described above. Polyethylene glycol-mediated DNA transfection was performed as previously described [27]. The protoplast suspension (2.0 × 10^4^ protoplasts in 100 µL) was supplemented with a mixture of vectors carrying PDF1.2P::*Fluc*::NOST or GEA6P::*Fluc*::NOST with reference (35SP::*Renilla luciferase* [*Rluc*]::NOST) vector, additionally with or without 35SP::*JUL1*::V5::NOST, 35SP::*JAV1*::3 × FLAG::NOST, 35SP::*JiTF*:: 4 × Myc::NOST at a ratio of 4:1(:5:5) to protoplast suspension with 110 µL of PEG solution [40% (w/v) polyethylene glycerol, 0.6 M mannitol, and 15 mM Ca(NO_3_)_2_4H_2_O]. The transfection was carried out at room temperature for 15 min and stopped by adding 400 µL of W5 solution. The protoplasts were collected by centrifugation at 100× *g* for 2 min and resuspended with 500 µL of WI solution (5 mM MES [pH 5.7], 0.6 M mannitol, and 20 mM KCl) and incubated at room temperature overnight. 

To confirm transient expression of genes in protoplasts, the transcriptional products in Arabidopsis protoplasts were evaluated by reverse transcription (RT)-PCR using ReverTra Ace^®^ (Toyobo, Osaka, Japan) and quantitative PCR (qPCR) (see below) (Figure S4). Three independent assays for protoplasts prepared from independent batches of Arabidopsis leaf samples were evaluated.

### 4.6. Luciferase (LUC) Assay

The LUC assay was performed as previously described [25]. Fluc activity produced due to the transfected reporter construct was expressed as the value normalized by the Rluc activity produced due to the cotransfected reference vector. Replicate analyses were conducted with 5 independent samples.

### 4.7. RNA Extraction, cDNA Synthesis and qPCR

Approximately 100 mg of Arabidopsis leaf tissues and 2.0 × 10^4^ protoplasts (see above) were homogenized in liquid nitrogen, and total RNA was isolated and purified using Sepasol^®^-RNA I Super G (Nacalai Tesque, Kyoto, Japan) following the manufacturer’s protocol. First-strand cDNA was prepared and qPCR was performed according to the method described previously [28]. Relative transcript abundances were determined after normalization of raw signals with the abundance of the actin gene (*AtACT8* [At1g49240]). We did not use samples or data when sufficient amounts of RNA (>83 ng µL^−1^) were not isolated from leaves or when abnormal quantification cycle (Cq) values for the actin gene were obtained.

### 4.8. BiFC

The full-length ORFs of *JUL1*, *JAV1* and *JAZ8* were inserted into pDEST-GW-nVenus (35SP::GW::N-terminal fragment of Venus (nVenus)::NOST], resulting in the construction of vectors JUL1-nVenus, JAV1-nVenus and JAZ8-nVenus, respectively. Likewise, the full-length ORFs of *JiTFs* (*ERF15*, *bZIP53* or *ORA59*) were inserted into pDEST-GW-cVenus (35SP::C-terminal fragment of Venus::GW::NOST], resulting in the construction of vectors JiTF-cVenus. These vectors were transformed into *Agrobacterium tumefaciens* EHA105 strain by electroporation.

A pair of *A. tumefaciens* cells carrying the individual vectors (JUL1-nVenus and JiTF-cVenus, JAV1-nVenus and JiTF-cVenus or JAZ8-nVenus and JiTF-cVenus) were pressure-infiltrated into the leaves of *Nicotiana benthamiana* plants. The transformed plants were incubated for 24 h in a climate-controlled room at 24 ± 1 °C, and then 50 µM MG132 (a proteasome inhibitor; FUJIFILM Wako Pure Chemical Corporation, Ltd.) in 0.1% dimethyl sulfoxide was applied to prevent protein degradation via the ubiquitin–proteasome system [29]. The transformed plants were additionally incubated for 24 h in a climate-controlled room at 24 ± 1 °C, and the fluorescence was observed under a BZ-X700 fluorescence microscope (Keyence Co., Osaka, Japan). For staining the nucleus, the sample was pretreated with 0.2 mM 4’,6-diamidino-2-phenylindole (DAPI) (FUJIFILM Wako Pure Chemical Corporation, Ltd.) for 5 min before observation.

### 4.9. In Vitro Ubiquitination Assay

The full-length ORFs of *ERF15*, *bZIP53*, *ORA59* and *JAV1* cDNA clones were inserted into the GW vector pEU-bls-GW. Likewise, the full-length ORF of *JUL1*, *GFP* or *ERF15* cDNA clones (without stop codon) was inserted into the GW vector pEU-GW-AGIA. Cell-free protein synthesis and in vitro ubiquitination assay were carried out according to the methods described previously [30]. Substrates were precipitated using FG beads which conjugated with streptavidin (TAMAGAWA SEIKI Co., Ltd. Nagano, Japan). Proteins were subjected to 8% SDS-PAGE and immunoblotted with anti-FLAG M2-peroxidase antibody produced by mouse clone M2 (Sigma-Aldrich) and HRP-linked anti-mouse antibody (Cell Signaling Technology). The signals were detected with an ImageQuant LAS-4000 imaging system (see above).

### 4.10. Statistical Analyses and Data Reproducibility

We performed one-way ANOVA with Holm’s sequential Bonferroni post hoc test or post hoc Tukey’s HSD using the program (http://astatsa.com/OneWay_Anova_with_TukeyHSD/ (accessed in 1 November 2022)). The sample sizes and number of replicates for all of the sets of assays and analyses are indicated in the legends of the corresponding figures.

## 5. Conclusions

Although the current study demonstrated transcriptional reprogramming of non-WRKYs by the JAV1/JUL1 system, the effects of the JAV1/JUL1 system on many other JiTFs remain to be addressed. Moreover, why JUL1 relies on ERF15 as a scaffold is still a matter of speculation. In this regard, comprehensive transcriptional analysis using *JiTF* and *JUL1* mutant plants to identify regulated genes other than *PDF1.2* and *GEA6* will further our understanding.

## Supplementary Materials

The following supporting information can be downloaded at: https://www.mdpi.com/article/10.3390/ijms24020987/s1.
