# Supplementary material for: JUL1, Ring-Type E3 Ubiquitin Ligase, Is Involved in Transcriptional Reprogramming for ERF15-Mediated Gene Regulation
Source: Int J Mol Sci. 2023 Jan 4;24(2):987. doi: 10.3390/ijms24020987 (PMC9863049; doi:10.3390/ijms24020987)
Supplement: Supplementary file 1 [file ijms-24-00987-s001.zip › Supplementary FIgures.pdf]

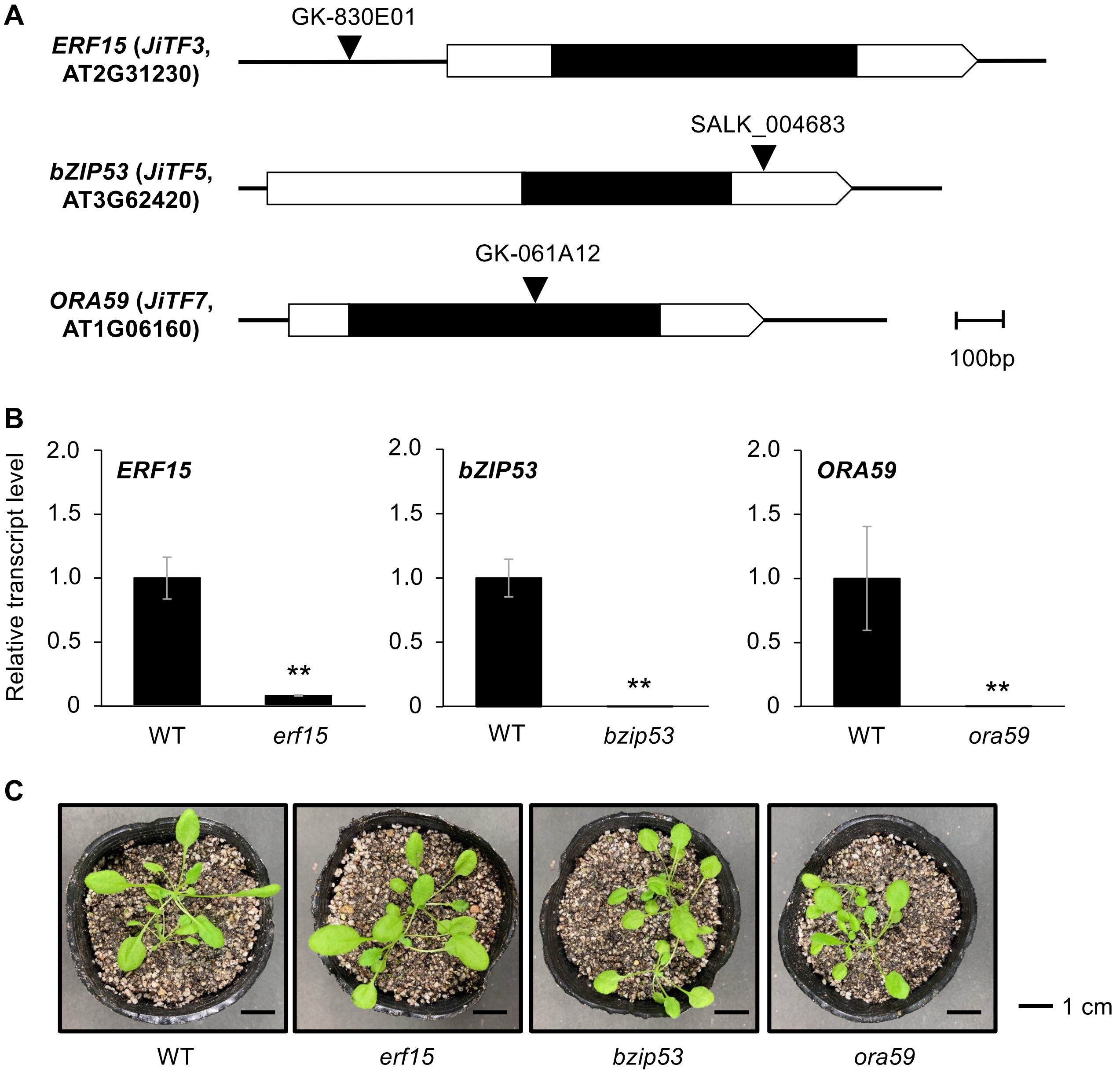
A
GK-830E01
ERF15 (JiTF3, AT2G31230)
SALK_004683
bZIP53 (JiTF5, AT3G62420)
GK-061A12
ORA59 (JiTF7, AT1G06160)
100bp
B
Relative transcript level
ERF15
bZIP53
ORA59
2.0
1.5
1.0
0.5
0
**
WT
erf15
bzip53
ora59
C
1 cm
WT
erf15
bzip53
ora59

Figure S1

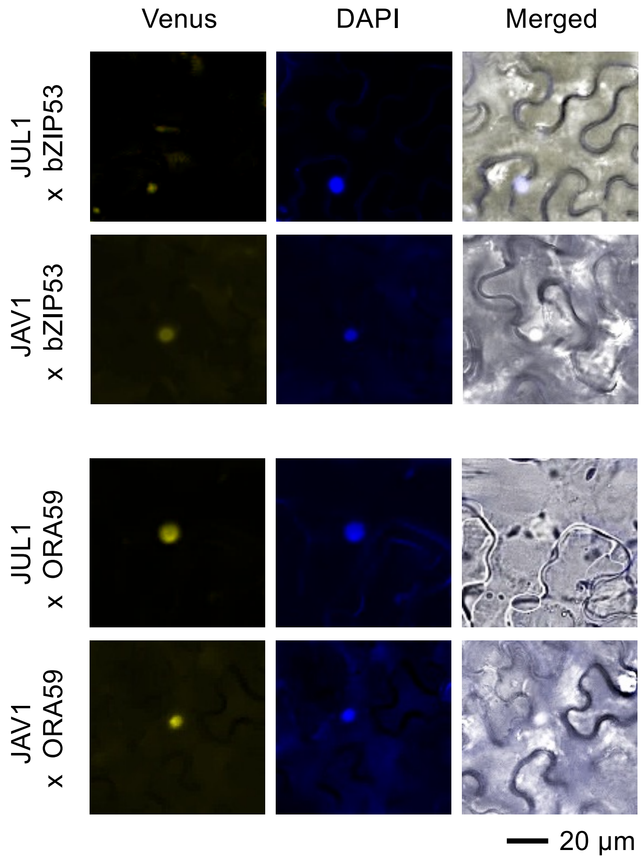

Venus
DAPI
Merged
JUL1 x bZIP53
JAV1 x bZIP53
JUL1 x ORA59
JAV1 x ORA59
20 µm

Figure S2

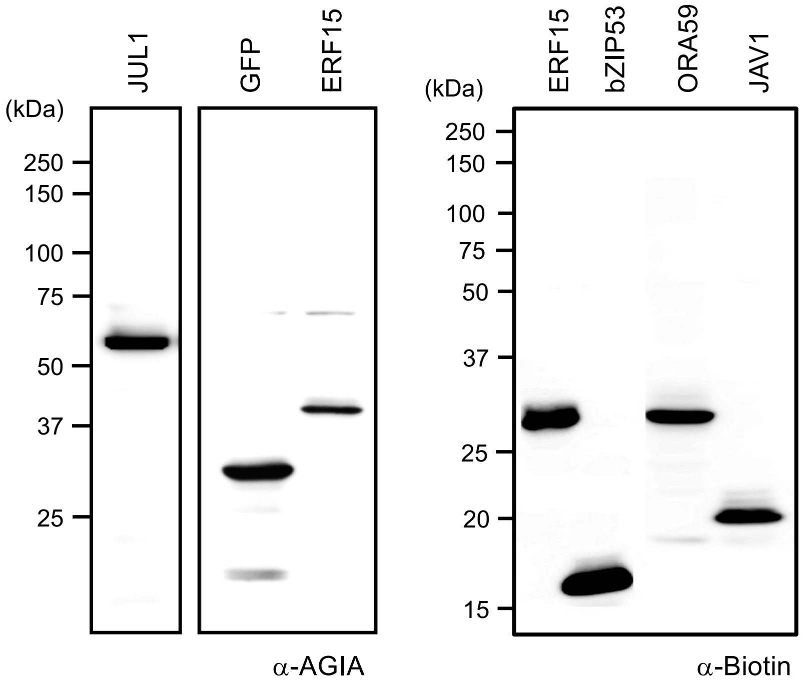
JUL1
GFP
ERF15
(kDa)
250
150
100
75
50
37
25
α-AGIA
ERF15
bZIP53
ORA59
JAV1
(kDa)
250
150
100
75
50
37
25
20
15
α-Biotin

Figure S3

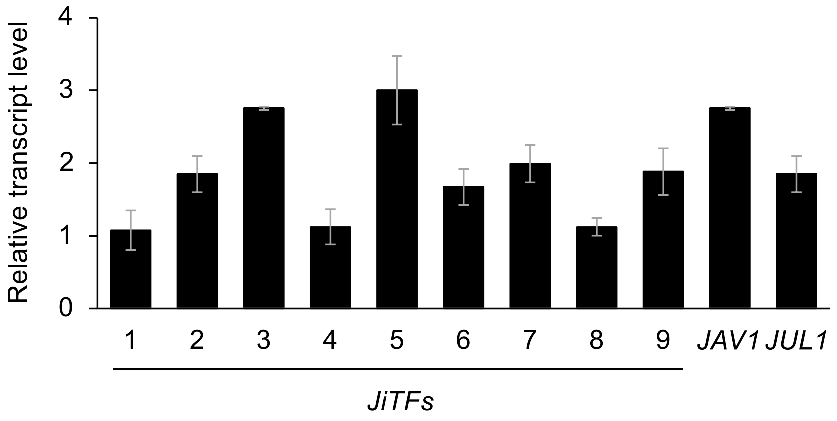
Relative transcript level
4
3
2
1
0
1
2
3
4
5
6
7
8
9
JAV1
JUL1
JiTFs

Figure S4

Figure S1. Basic phenotypes of the JiTF mutants. (A) T-DNA insertion sites in *erf15* (GK-830E01), *bzip53* (SALK_004683) and *ora59* (GK-061A12). White and dark boxes indicate untranslated regions and translated regions, respectively. Dark areas and white areas indicate open reading frames and untranslated regions of genes, respectively. (B) Relative transcript levels of *ERF15*, *bZIP53* and *ORA59* in leaves of the respective mutants and Arabidopsis wild-type (WT) plants. Data represent the mean and standard error (n = 6). Data marked with asterisks are significantly different from those in WT, based on an ANOVA with Holm's sequential Bonferroni post-hoc test ($P < 0.01$). (C) Potted plants 4 weeks after planting.

Figure S2. *In planta* interaction between JUL1 and bZIP53 or ORA59 and between JAV1 and bZIP53 or ORA59. Bimolecular fluorescence complementation analysis of *in planta* interaction of JUL1 or JAV1fused to the N-terminal fragment of Venus with bZIP53 or ORA59 fused to the C-terminal fragment of Venus in *Nicotiana benthamiana* leaf cells. The photographs with reconstructed Venus signal, DAPI (4′,6-diamidino-2-phenylindole) fluorescence, and the merged image with bright field are shown.

Figure S3. Immunoblotting of proteins synthesized using the cell-free system. Total proteins were subjected to 10% SDS-polyacrylamide gel electrophoresis and immunoblotted with the indicated antibody (α-AGIA or α-biotin).

Figure S4. Transient expression levels of *JiTFs*, *JAV1*, and *JUL1* in protoplasts. Quantitative transcript abundances relative to those of *AtACT8*, based on 3 independent assays for protoplasts prepared from independent batches of *Arabidopsis thaliana* leaf samples were assessed. Data represent the mean and standard error.
